# Maternal probiotics intake during pregnancy and exclusive colostrum breastfeeding are associated with a reduced risk of neonatal jaundice

**DOI:** 10.1186/s12916-025-04516-x

**Published:** 2025-12-08

**Authors:** Bekalu Kassie Alemu, Chi Chiu Wang, Liona C. Poon, Yao Wang

**Affiliations:** 1 Department of Obstetrics and Gynaecology, The Chinese University of Hong Kong, 1/F, Special Block E, Prince of Wales Hospital, 30-32 Ngan Shing Street, Shatin NT, Hong Kong, Special Administrative Region China; 2 Department of Midwifery, College of Medicine and Health Sciences, Debre Markos University, Debre Markos, Ethiopia; 3 Li Ka Shing Institute of Health Sciences, Faculty of Medicine, The Chinese University of Hong Kong, Hong Kong, Special Administrative Region China

**Keywords:** Neonatal jaundice, Exclusive breastfeeding, Probiotics, Colostrum, Modifiable lifestyle intervention, Fetal health, Microbiome, CHILD cohort, Cohort study

## Abstract

**Background:**

Neonatal jaundice (NJ), characterized by significantly increased bilirubin levels, is a prevalent global neonatal condition affecting 60–80% of newborns. It imposes long-term adverse effects on neurodevelopment and overall health. Current clinical treatments, such as phototherapy, primarily focus on symptom management, whereas the preventive strategies for NJ remain largely lacking. Infant breastfeeding is associated with NJ. However, whether maternal probiotics use and infant colostrum feeding may reduce the NJ risk has yet to be determined and warrants further investigation in large-scale cohorts. Therefore, this study aimed to evaluate whether they have any preventive effect.

**Methods:**

We investigated the relationship of maternal probiotic intake and baby feeding type with NJ onset using the CHILD cohort, a prospective birth cohort recruited 3624 mothers and 3542 paired infants. Probiotic intake during pregnancy and its patterns (increased, maintained, or decreased compared to preconception) were obtained via questionnaires. The NJ conditions (yes/no) and feeding modes (exclusive colostrum, formula-only, or mixed feeding) were collected from hospital birth records. Bivariate and multivariable logistic regressions were employed to evaluate the risk using adjusted odds ratio (AOR) with 95% confidence intervals (CI) after adjustment for confounders. *P* < 0.05 indicates statistical significance.

**Results:**

A total of 2596 healthy controls and 433 NJ cases with complete data were included from the CHILD cohort for analysis. Interestingly, probiotic intake during pregnancy was associated with a remarkably reduced odds of NJ (AOR 0.78 (0.61, 0.98), *P* = 0.041) compared to participants who never used probiotics. Neonates with exclusive colostrum feeding also had a significantly lower incidence of NJ than other feeding modes (AOR 0.34 with (95%CI) (0.27,0.44), *P* < 0.001). Further stratification analysis on probiotic intake showed that mothers who increased (AOR 0.53 (0.32, 0.89), *P* = 0.016) or maintained (AOR 0.44 (0.24, 0.80), *P* < 0.007) probiotic intake during pregnancy had a lower risk of NJ compared to those who decreased intake.

**Conclusions:**

This cohort-based evidence highlights that maternal probiotic intake and exclusive colostrum feeding are important modifiable factors associated with reduced NJ risk.

**Graphical Abstract:**

Summary of the association of maternal probiotics use and exclusive colostrum feeding with neonatal jaundice (using the data from the CHILD longitudinal birth cohort). The figure is created using BioRender.com. CHILD, Canadian Healthy Infant Development; n, number of participants involved in the corresponding sections.

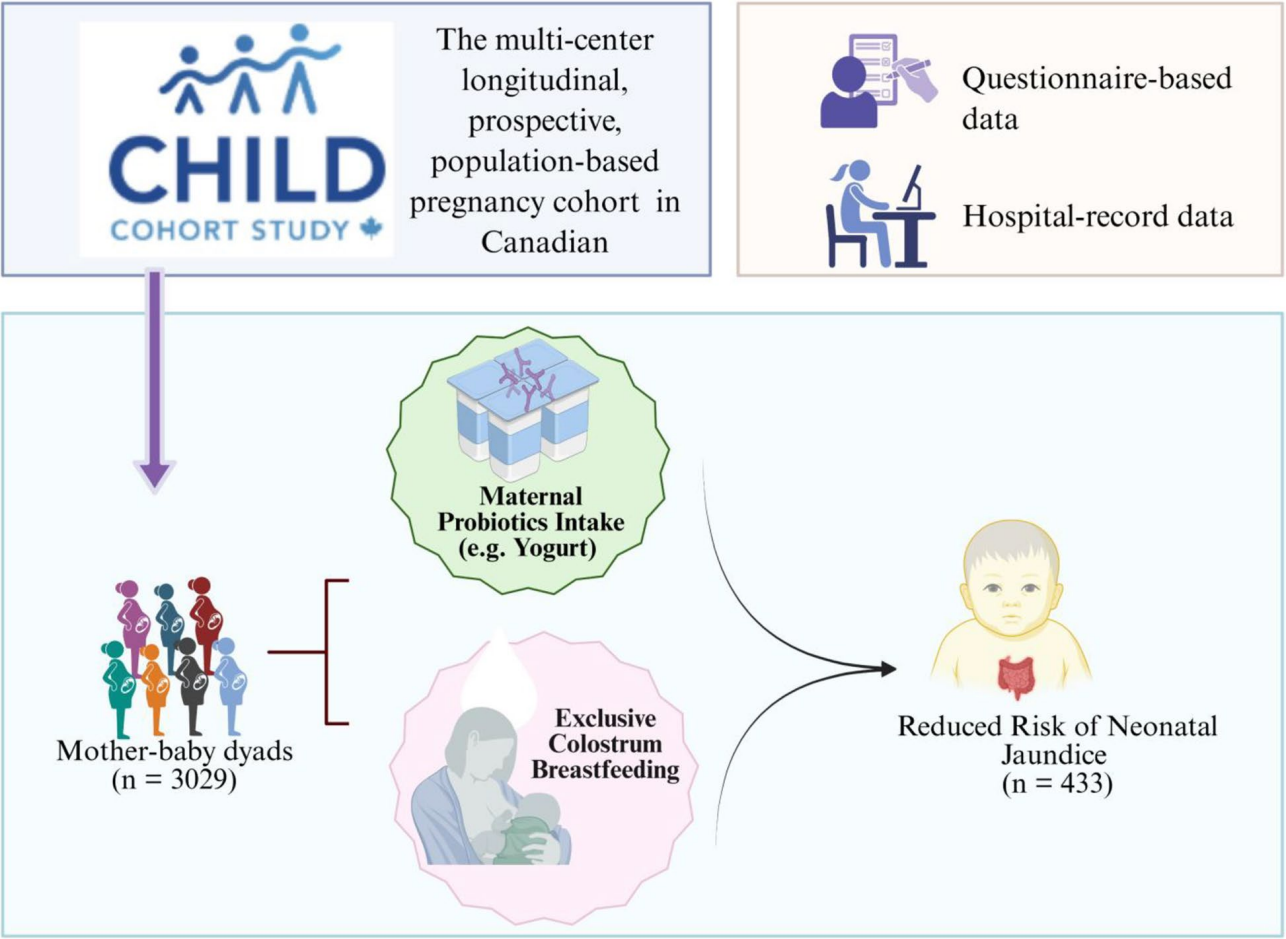

**Supplementary Information:**

The online version contains supplementary material available at 10.1186/s12916-025-04516-x.

## Background

Neonatal jaundice (NJ) is characterized by a yellowish discoloration in the skin, sclera, and mucous membrane of newborns due to elevated circulating bilirubin levels [1–3]. It affects more than 60% of full-term and 80% of preterm newborns [1, 4–6]. The pathologic NJ emerges within the first 24 h with rapidly increased total serum bilirubin (TSB) level (≥ 5 mg/dL/day or > 0.2 mg/dL/h) and often lasts beyond 14 days, whereas the physiologic type mostly occurs after 24 h of life and fades out within 2 weeks [2, 7, 8]. Severe NJ (TSB > 15 mg/dL) potentially leads to bilirubin encephalopathy (BE, an acute phase of bilirubin neurotoxicity), bilirubin-induced neurologic dysfunction (BIND, a chronic sequelae of bilirubin exposure) [9], permanent brain damage, or even death [10]. Importantly, it also contributes to cardiac rhythm disturbances, dehydration, hypocalcemia, and renal damage [11]. While the causal relationship between NJ and long-term developmental disorders and neurodevelopmental outcomes remains to be investigated [12], NJ may pose chronic detrimental effects on an infant’s health, including developmental delays, infantile cerebral palsy, speech disorders, dysarthria, intellectual disabilities, and autism [13]. Currently, phototherapy and exchange transfusion are the primary management for NJ [6]. However, they exhibit potential side effects on vision impairment, hearing problems, and metabolic acidosis [4, 14]. Therefore, there is an urgent need to develop novel preventive strategies for NJ.

The early life microbiome is pivotal in determining the lifelong health trajectory [15, 16], including brain development, immune imprinting, and maturation, and gut development [17, 18]. Human gut microbiota colonization is initially established by maternal–fetal transmission [15], starting in utero during pregnancy, followed by exposure through the birth canal at delivery, and subsequently shaped by breastfeeding [19]. This vertical maternal microbiome inheritance is influenced by multiple perinatal factors, including feeding type, gestational age, and delivery mode [20]. In addition, maternal factors such as nutrition and pharmacological exposure also affect mother–offspring microbiome interplay [19]. Maternal microbiota-targeted interventions, mainly probiotics, modulate the infant gut microbiome by increasing beneficial bacteria abundance [21]. Therefore, the perinatal period emerges as a critical window for shaping infant health trajectories by establishing a resilient and functional gut microbiota.

Breastfeeding is a behavioral intervention with well-documented maternal-infant benefits and strongly recommended by global health authorities. It mitigates the risk of infection, supports neurocognitive development, and maintains the overall health of infants [22–24]. Breast milk (BM) contains immunoglobulins, bioactive molecules, and a complex microbial community, functioning as both nutritional and microbial vectors [25]. Growing studies have shown that the BM microbiome shapes the infant microbiome, regulates early life immunity, and prevents diseases such as atopic dermatitis [25, 26]. Of note, it can be modulated through maternal microbiome-targeted interventions, particularly probiotics supplementation [26]. Maternal probiotic intake effectively increases the abundance of beneficial bacteria such as *lactobacilli* and *bifidobacteria*, while reducing the pathogenic bacteria load, mainly *staphylococci* in BM, and further remodels the gut microbiome composition in offspring [26]28. Hence, BM serves as a vital mediator regulating the maternal-infant microbiome transmission, thereby contributing to neonatal development and lifelong health [25, 27].

Interestingly, accumulating evidence suggests that NJ is associated with microbiome dysbiosis [28], as evidenced by a lower abundance of *Bacteroides* and *Bifidobacteria* in neonates with NJ compared to healthy infants [29]. Bilirubin excretion and clearance require the transformation of conjugated bilirubin into stercobilin and urobilin [30, 31], which is catalyzed by bacterial bilirubin reductase enzyme and β-glucuronidase [32]. Dysfunction in this process enhances enterohepatic bilirubin resorption, thereby leading to hyperbilirubinemia and jaundice.

Therefore, given the well-known benefits of maternal probiotics and breastfeeding in modulating the neonatal microbiome, we hypothesized whether they may affect the incidence of NJ. To this end, we investigated their potential association using data from the CHILD Cohort Study [33], an internationally recognized, population-based longitudinal birth cohort of over 3500 mother-infant pairs.

## Methods

### Study design, setting, and participants

This study was based on participants in the CHILD Cohort Study, a prospective longitudinal birth cohort that was conducted between 2008 and 2012 at four Canadian health centers (Edmonton, Toronto, Manitoba, and Vancouver). A total of 3624 pregnant women were initially recruited, and 3542 infant pairs were subsequently eligible for follow-up [33]. Mothers who met the following criteria were enrolled in the cohort: age > 18 years, living within 50 km of a participating delivery hospital, and planned to give birth at a recruitment hospital, English speaker, and the infant was born at ≥ 35 weeks. Exclusion criteria included those who gave birth to a baby with respiratory distress syndrome or major congenital abnormalities, multiple births, or those resulting from in vitro fertilization [34]. Data was collected from 18 weeks of gestation until childhood and later. Following the Declaration of Helsinki, written informed consent was obtained from each mother [35]. The cohort collected questionnaire-based data, clinical data from hospital records, took measurements and laboratory tests, and collected biological samples for further analysis.

### Outcome measures

The primary outcome of this study was the occurrence of NJ and its relationship with maternal probiotics intake and colostrum feeding. The NJ status (yes/no) and feeding modes (exclusive colostrum, formula feeding, or mixed feeding) were collected from hospital records, while probiotic (e.g., yogurt) intake data (increased intake, maintained, or reduced intake during pregnancy) were collected using a questionnaire. The associations of maternal clinical factors, such as pregnancy complications (e.g., preeclampsia) and treatment characteristics like antibiotic use, were modeled as secondary outcomes.

### Eligibility criteria for this study

Sociodemographic and clinical data of maternal-infant pairs were extracted from the CHILD database. For inclusion in the analysis, neonatal jaundice (NJ) status must be recorded as “YES/NO.” Similarly, probiotics intake data should be categorized as “YES/NO” based on the following classifications: never, prior to pregnancy, during pregnancy, increased intake, continued intake without changes, or decreased intake during pregnancy.

### Variables and definitions

**Fig. 1:**
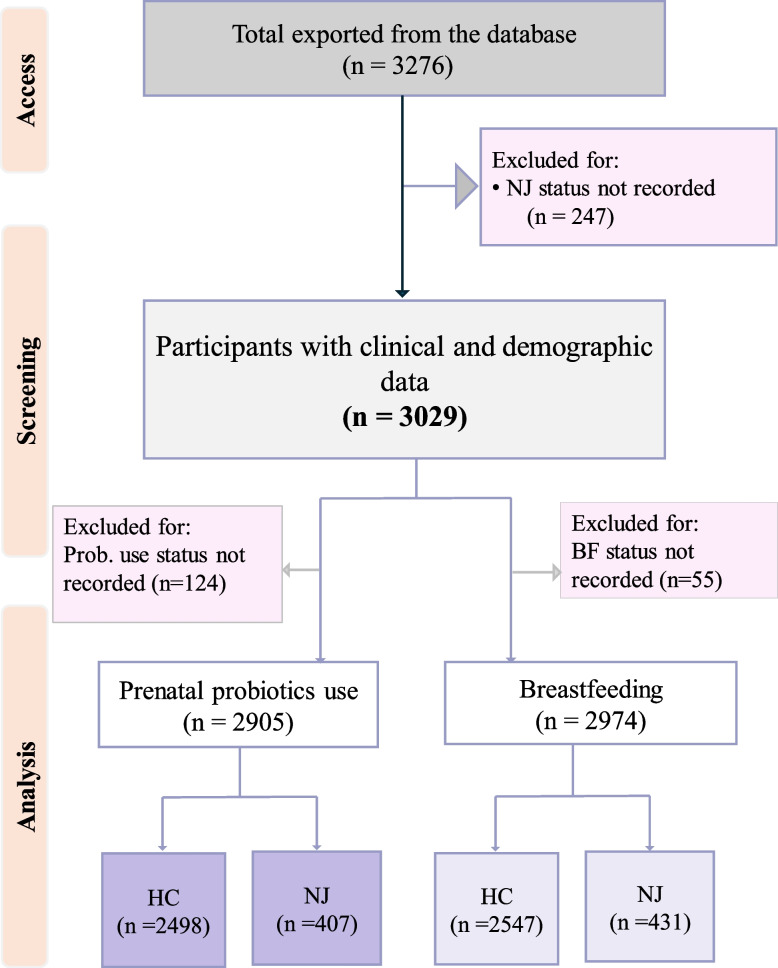
Data access, screening, and analysis flow diagram. HC, healthy controls; NJ, neonatal jaundice; *n*, number of participants. A CONSORT flow diagram is used to present the process from data access to the screening and analysis stages

Neonatal jaundice was the dependent variable reported as “YES” for jaundiced cases, while “NO” for healthy neonates. Probiotic intake in pre-pregnancy and prenatally was considered the primary determinant factor. Participants were asked whether they had taken probiotics, such as yogurt, during the 12 months before they became aware of their pregnancy or during pregnancy itself. Responses were categorized as follows: if no probiotics were consumed during either period, the participant was instructed to select “NEVER.” If probiotics were taken only during the 12 months before pregnancy recognition, “PRIOR” was selected. If intake occurred only during pregnancy, “PREG” was marked. For participants who consumed probiotics both before and during pregnancy, both “PRIOR” and “PREG” were selected, along with an indication of any change in intake pattern during pregnancy: “INC” for increased intake, “SAME” for no change, and “DEC” for decreased intake. At the same time, maternal characteristics, clinical outcomes of mother and baby, and obstetric outcomes were taken as covariates adjusted in the analysis models. BM is classified as colostrum (0–4 days), transitional milk (5–14 days), and mature milk (after 2 weeks) [36–38]. Following the approval of the concept proposal, our team has been granted access to all participants’ (mother-infant pairs) data, and finally, we have used more than 3000 paired participants as detailed in the CONSORT flow diagram (Fig. 1).

### Data access from the CHILD database

To access the data, we have passed several relevant approval steps. We developed and submitted a concept proposal to the database management bodies after obtaining approval for ethics from CREC. An agreement on data and sample transfer (DSTA) was established between McMaster University (the provider institution) and the Department of Obstetrics and Gynaecology of the Chinese University of Hong Kong and signed by authorized representatives of both institutions as well as the CHILD Cohort Study Director and the Principal Investigator (PI) of this study. Both parties met the administrative requirements, and the research team was granted access to the data. The PI was responsible and allowed to download the Default Data Export file. The file was exported for further data processing and analysis.

### Data processing and analysis

The data were exported in an Excel file with a linked legend file that clearly showed the definition, coding, question, and category study variables (Additional file: Table S1). The data for responses such as not applicable, participant skipped the question, not recorded, and questionnaire not applicable were cleaned. Continuous data were also transformed into clinically meaningful categories. In consultation with the bioinformatician in the CHILD database team, we cleaned the “0” values entered for the patterns of probiotics use (“Increase,” “No change,” and “Decrease”) when the “Never” use was marked as “Yes” because of its inappropriateness to code as “0” which means “No” (Additional file: Table S2).

Socio-demographic and clinical characteristics were summarized in percentages and mean with standard deviations (SD) and presented in a table. We compared clinical factors and sociodemographic variables using Pearson’s chi-square test and independent *t*-tests. A binary logistic regression model was utilized to assess the relationship between neonatal jaundice, maternal probiotic intake, and the type of baby feeding. Variables with a *P*-value less than 0.25 in the bivariate analysis were included in the multivariable logistic regression analysis. The statistical significance was declared when the *P*-value was < 0.05. The findings were presented in texts, tables, and forest plots. The strength of the association was measured using adjusted odds ratios (AOR). Stata 17 software (StataCorp LLC, 4905 Lakeway Drive, College Station, TX 77845, USA) was used to analyze the data. Forest plots of the multivariable logistic regression analysis were generated using GraphPad Prism 8 for Windows (Version 8.0.2 (263), January 30, 2019).

## Results

### Participants’ demographic and clinical characteristics

A total of 2596 healthy controls and 433 jaundiced neonates were nested from the CHILD cohort for analysis (Fig. 1). The demographic information is stated in Table 1. As shown in the table, most (62.4%) of mothers included in the analysis were multiparous (*n* = 1889), attended university and above education (70.5%, *n* = 2048), and employed (71.9%, *n* = 2068). There were significant differences in the maternal (mean ± SD) (in NJ, 31.6 ± 4.7; and in healthy controls/HC, 32.3 ± 4.7,* P* = 0.004) and paternal ages (NJ, 33.8 ± 5.6 years; HC, 34.4 ± 5.4, *P* = 0.036). The significant difference was also observed in employment status (*P* = 0.004), parity (*P* = 0.002), and mode of delivery (*P* < 0.001) (Table 1). Then, we further compared the incidence rate of major gestational complications in the two groups, including antepartum bleeding, prelabor nausea, infection, preeclampsia (PE), and gestational diabetes (GDM). Mothers in NJ groups exhibited a significantly increased PE (*P* = 0.001) and GDM (*P* = 0.009). This difference was also demonstrated in the participants’ intrapartum antibiotics use, where NJ groups took in a significantly (*P* < 0.001) higher proportion (54.1%) than HC (42.3%) (Table 2). Cesarean delivery in the NJ group was higher (33.3%) than in the HC group (23.7%) (*P* < 0.001).

**Table 1:** Mothers’ sociodemographic and clinical characteristics

Variables (*n* = NJ/J)	HC (*n* = 2596)	Jaundiced (*n* = 433)	*P*-value
Maternal age (years)	32.3 ± 4.7	31.6 ± 4.7	0.004
Father’s age (years)	34.4 ± 5.4	33.8 ± 5.6	0.036
Mother’s highest education (*n* = 2496/407)			
High school or below	210 (8.4)	45 (11.1)	0.226
College	510 (20.4)	90 (22.1)	
University	1298 (52.0)	202 (49.6)	
Master or PhD	478 (19.2)	70 (17.2)	
Father’s highest education (*n* = 2474/404)			
High school or below	365 (14.7)	73 (18.1)	0.193
College	638 (25.8)	112 (27.7)	
University	1076 (43.5)	158 (39.1)	
Master or PhD	395 (16.0)	61 (15.1)	
Maternal employment status (*n* = 2146/367)			
Employed	2068 (96.4)	327 (89.1)	< 0.001
Non-employed	78 (3.6)	40 (10.9)	
Parity (*n* = 2595/433)			
Primipara	943 (36.3)	196 (45.3)	0.002
Multipara	1489 (57.4)	213 (49.2)	
Grand multipara	163 (6.3)	24 (5.5)	
Gestational diabetes	116 (4.5)	32 (7.4)	0.009
Preeclampsia	80 (3.1)	27 (6.2)	0.001
Prelabor bleeding	178 (6.9)	31 (7.2)	0.818
Prelabor nausea	530 (20.4)	100 (23.1)	0.204
Prelabor infection	85 (3.3)	16 (3.7)	0.652
Mode of delivery (*n* = 2596/432)			
Spontaneous vaginal	1691 (65.1)	233 (53.9)	< 0.001
Assisted vaginal	289 (11.1)	55 (12.7)	
Cesarean section	616 (23.7)	144 (33.3)	
Baby sex			
Male	1353 (52.1)	247 (57.0)	0.057
Female	1243 (47.9)	186 (43.0)	
Baby birth weight (gms), (*n* = 2582/430)	3455.2 ± 468.1	3347.2 ± 537.7	< 0.001
< 2500	52 (2.0)	27 (6.3)	
2500–3999	2206 (85.4)	356 (82.8)	
≥ 4000	324 (12.6)	47 (10.9)	
Gestational age (weeks), (*n* = 2595/430)	39.2 ± 1.3	38.7 ± 1.7	< 0.001
Preterm	84 (3.2)	43 (10)	
Term	2511 (96.8)	387 (90.0)	
Baby body length (cm)	51.5 ± 2.6	51.3 ± 2.6	0.336
Baby head circumference (cm)	34.6 ± 1.4	34.5 ± 1.5	0.406
First min Apgar (*n* = 2580/426)	2345 (90.9)	367 (86.2)	0.002
Fifth min Apgar (*n* = 2580/426)	2547 (98.7)	421 (98.8)	0.857
Any baby abnormality (*n* = 2516/410)	151 (6.0)	48 (11.7)	< 0.001
Duration of ROM (hrs)	13.9 ± 7.7	13.5 ± 7.4	0.415

Categorical variables are presented in *n* (%) and continuous variables in mean ± SD

*(n* = *NJ/J)* sample size of non-jaundiced/jaundiced cases in respective variables, *gms* grams, *cm* centimeters, *ROM* rupture of membrane, *hrs* hours

*Primipara* a woman who has given birth once, *Multipara* two to four times, *Grand multipara* five or more times at ≥ 20 weeks of gestation

**Table 2:** Comparison of maternal probiotics and antibiotics use between jaundiced neonates and healthy controls

Variables (*n* = NJ/J)	Healthy controls (*n* = 2596)	Jaundiced (*n* = 433)	*P*-value
Intrapartum antibiotics use (*n* = 2570/431)	1086 (42.3)	233 (54.1)	< 0.001
Ever use of probiotics (*n* = 2346/409)	1291 (55.0)	201 (49.1)	0.0270
Probiotics intake prior to pregnancy	1291 (74.5)	224 (53.1)	< 0.001
Probiotics intake during pregnancy	1261 (72.8)	202 (47.9)	< 0.001
Prior to + during pregnancy			
Increase intake during pregnancy	268 (19.5)	33 (15.0)	0.111
Maintain intake during pregnancy	853 (62.2)	116 (52.7)	0.008
Decrease intake during pregnancy	106 (7.7)	30 (13.6)	0.004
Breastfeeding in hospital (*n* = 2547/431)	2470 (97.1)	416 (96.5)	0.490
Baby’s feeding type (*n* = 2111/410)			
Exclusive colostrum feeding	1544 (73.1)	186 (45.4)	< 0.001
Breast + formula	510 (24.2)	210 (51.2)	
Formula feeding only	57 (2.7)	14 (3.4)	

Variables are presented in *n* (%)

*(n* = *NJ/J)*, sample size of non-jaundiced/jaundiced cases in respective variables

Regarding infant outcomes, babies were assessed for any abnormalities in the immediate postpartum period (Table 1). It was found that 11.7% of NJ and 6.0% of HC cases were diagnosed with abnormalities (*P* < 0.001). Preterm birth was 10% in NJ and 3.2% in the HC groups. Moreover, the first-minute Apgar scores (Appearance (skin color), Pulse (heart rate), Grimace (reflex response), Activity (muscle tone), and Respiration (breathing)) [39] were significantly differed in the two groups (*P* = 0.002). NJ is reported to have a low body weight in grams (3347.2 ± 537.7) compared to HC (3455.2 ± 468.1, *P* < 0.001). Consistently, we found that the birth weight of the jaundiced group was significantly lower (38.7 ± 1.7) than that of HC (39.2 ± 1.3, *P* < 0.001).

### Preeclampsia and prelabor nausea are major determinants of neonatal jaundice

**Fig. 2:**
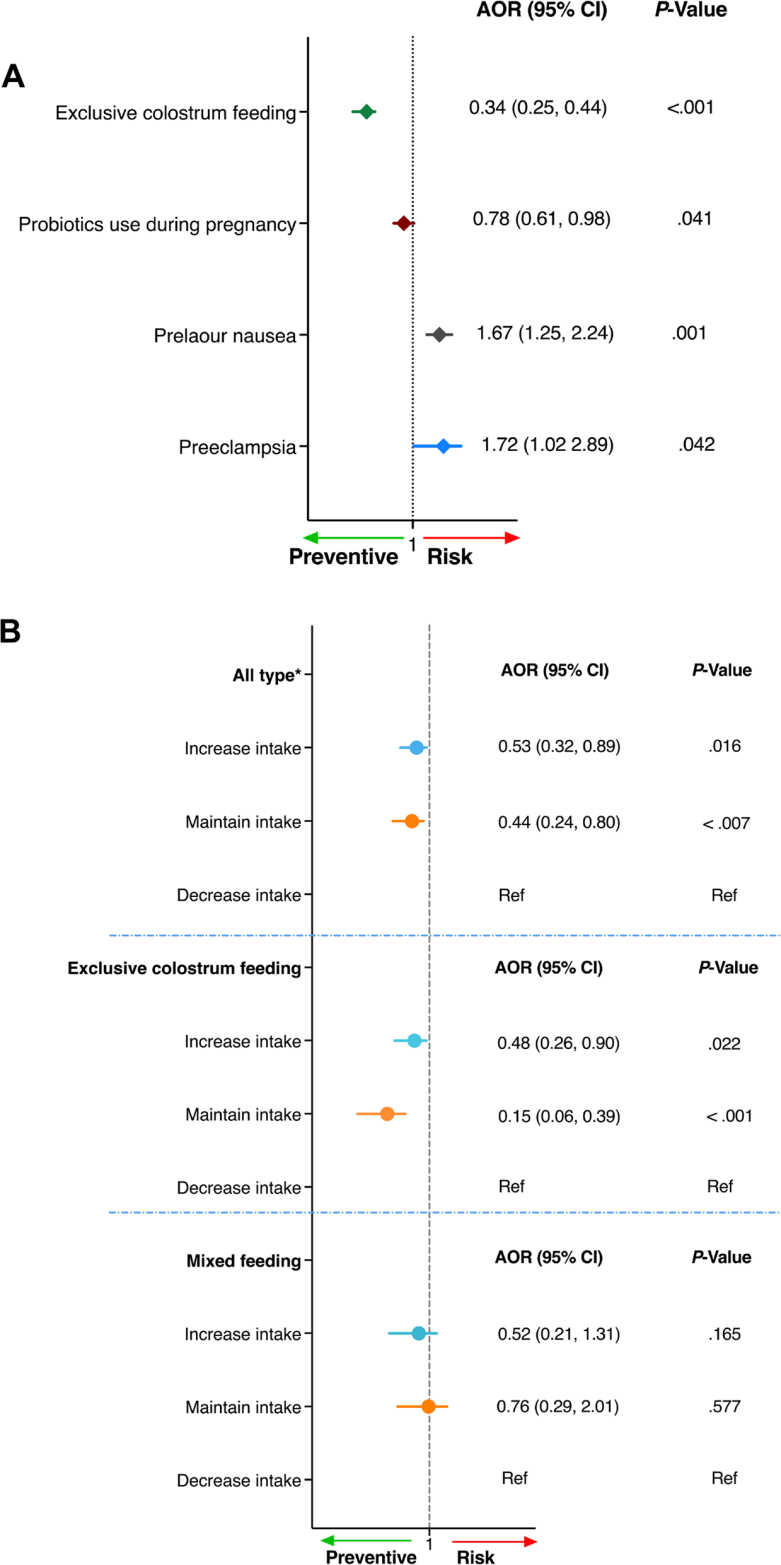
Exclusive colostrum feeding and maternal probiotics intake are associated with reduced risk for neonatal jaundice.** A** Determinant factors of NJ with a multivariable logistic regression analysis; The AORs were adjusted for parity, gestational diabetes, intrapartum antibiotics use, and mode of delivery. **B** A subgroup of association of maternal probiotics intake with neonatal jaundice by feeding type of babies in the hospital from the Canadian Healthy Infant Longitudinal Development (CHILD) Cohort Study. The AORs were adjusted for parity, prelabor nausea, preeclampsia, gestational diabetes intrapartum antibiotics use, mode of delivery, and gestational age at birth (in all type feeding); parity, prelabor nausea, preeclampsia, gestational diabetes, intrapartum antibiotics use, and baby sex (in exclusive colostrum feeding); and maternal employment, prelabor nausea, preeclampsia, gestational diabetes, birth weight, and baby’s sex (in mixed feeding) groups. Adjusted factors were evaluated for multicollinearity using variance inflation factor (VIF) and variables with VIF > 10 were excluded step by step from each model. The definition of probiotic intake modes and breastfeeding patterns are listed as follows. Never use, the participant used probiotics (e.g., yogurt) neither within 12 months before she knew she was pregnant nor during pregnancy; use prior to pregnancy, participants used probiotics within 12 months before she knew she was pregnant; use during pregnancy, participants used probiotics during pregnancy; increased use, participants used probiotics both prior to and during pregnancy and increased the intake during pregnancy compared to prior; no change (maintain) use, participants used probiotics both prior to and during pregnancy with no change in intake pattern; decreased use, participant used probiotics both prior to and during pregnancy but decreased the intake during pregnancy compared to prior; all type*, breast milk, formula, or other feeding including dextrose; exclusive colostrum feeding, feeding only breast milk in hospital (within 1–4 days after delivery); mixed feeding, feeding both breast milk and formula in the hospital

Since the two groups had significant differences in clinical profiles (Table 2), we first evaluated the major risk factors associated with NJ using multivariable logistic regression analyses. Potential confounding factors with *P* < 0.25 in the bivariable logistic regression analyses were included in the multivariable regression model, including parents’ education level, parity, preeclampsia, gestational diabetes, probiotics use during pregnancy, mode of delivery, intrapartum antibiotics use, 1 st minute Apgar score, birth weight, gestational age at birth, and baby feeding type in hospital after birth. To avoid the potential multicollinearity effect, variables with a variance inflation factor (VIF) > 10 were excluded stepwise. After data filtration, we revealed that prelabor nausea (AOR 1.67 with 95% CI 1.25, 2.24, *P* = 0.001) and preeclampsia (AOR 1.72 with 95% CI 1.02, 1.89, *P* = 0.042) were associated with increased odds of NJ (Fig. 2A). On the contrary, exclusive colostrum-feeding was linked with a reduced NJ risk by 66% (AOR 0.34 with 95% CI 0.25, 0.44, *P* < 0.001) compared to non-exclusive (either mixed or formula-fed) babies. Moreover, maternal probiotics use during pregnancy reduced the odds of NJ by 22% (AOR 0.78 with 95% CI 0.61, 0.98, *P* = 0.041) compared to those who never used (Fig. 2A) (Table 3 part A).

**Table 3:** Determinants of NJ in a longitudinal birth cohort with adjusted confounders

	Study variables		AOR (95% CI)	*P*-value
A. Factors associated with NJ using a multivariable logistic regression analysis				
Determinants of NJ	Parity	Primipara	Ref	Ref
		Multipara	0.78 (0.61, 1.01)	0.056
		Grand multipara	0.82 (0.48, 1.43)	0.491
	Prelabor nausea		1.67 (1.25, 2.24)	0.001
	Preeclampsia		1.72 (1.02, 2.89)	0.042
	Gestational diabetes		1.24 (0.76, 2.05)	0.390
	Probiotics use	Never used	Ref	Ref
		Used during pregnancy	0.78 (0.61, 0.98)	0.041
	Intrapartum antibiotics use		1.25 (0.95, 1.66)	0.112
	Mode of delivery	Spontaneous vaginal	Ref	Ref
		Assisted vaginal	1.125 (0.86, 1.84)	0.236
		Cesarean section	1.25 (0.91, 1.71)	0.168
	Baby feeding	Mixed feeding	Ref	Ref
		ECF	0.34 (0.27, 0.44)	< 0.001
B. Multivariable regression analysis of maternal probiotics intake pattern and risk of NJ				
All types of feeding n/N	Parity	Primipara	Ref	Ref
		Multipara	0.56 (0.38, 0.83)	0.003
		Grand multipara	0.92 (0.39, 2.15)	0.851
	Prelabor nausea		1.69 (1.08, 2.62)	0.020
	Preeclampsia		1.74 (0.79, 3.88)	0.169
	Gestational diabetes		0.90 (0.39, 2.05)	0.794
	Intrapartum antibiotics use		1.28 (0.83, 1.97)	0.260
	Mode of delivery	Spontaneous vaginal	Ref	Ref
		Assisted vaginal	1.53 (0.88, 2.64)	0.128
		Cesarean section	1.30 (0.80, 2.10)	0.290
	GA at birth	Preterm	Ref	Ref
		Term	0.31 (0.15, 0.63)	0.001
	Baby feeding type	Mixed feeding	Ref	Ref
		EC feeding	0.41 (0.28, 0.62)	< 0.001

*Ref* reference, *PHI* pregnancy-induced hypertension, *GDM* gestational diabetes mellitus, *ECF* exclusive colostrum feeding, feeding only BM in hospital (within 1–4 days after delivery); mixed feeding, feeding both BM and formula in hospital (within 1–4 days after delivery)

### Probiotic intake pattern during pregnancy is associated with lower NJ risk

Since maternal probiotic use was significantly associated with reduced NJ risk, we subsequently conducted a subgroup analysis to delineate how probiotic intake patterns contribute to the NJ risk reduction. There was a significantly higher proportion of probiotics use reported in the HC group (*P* = 0.027). Similarly, probiotics intake was also higher in HC versus NJ during the preconception period (74.5 versus 53.1%, *P* < 0.001) and during gestation (72.8% versus 47.9%, *P* < 0.001) (Table 2). Moreover, among participants who used probiotics prior to and during pregnancy, the probiotic intake patterns had a significantly different impact on NJ occurrence. Although the proportion of mothers who increased probiotic intake was comparable between groups (19.5% in HC vs 15.0% in NJ; *P* = 0.111), maintained probiotic intake was more commonly observed in the HC group compared to the NJ group (62.2 vs 52.7%; *P* = 0.008). Importantly, 13.6% of mothers in NJ groups reported a reduction in probiotic intake relative to their pre-pregnancy levels, which was remarkably higher than HC groups (7.7%, *P* = 0.004) (Table 1). We further performed a multivariable logistic regression. The parity, mode of delivery, maternal medical complications such as PE and GDM, maturity (GA at birth), and baby feeding type were fitted with the pattern of probiotics intake after exclusion of variables with higher VIF (> 10). We demonstrated that mothers who increased (AOR 0.53 (0.32,0.89), *P* = 0.016) and maintained (AOR 0.44 (0.24, 0.80), *P* < 0.007) probiotics intake during pregnancy were associated with a lower risk of NJ in comparison with those who decreased intake (Fig. 2B). Multiparity compared to primiparous (AOR 0.56 (0.38, 0.83), *P* = 0.003) and term babies relative to preterm ones (AOR 0.31 (0.15, 0.63), *P* = 0.001) also had lower odds of NJ. Pre-labor nausea, on the contrary, increased the odds of NJ by 31% (AOR 1.69 (1.08, 2.62), *P* = 0.020) (Table 3 part B). Taken together, our findings indicate that increased and maintained maternal probiotics intake is linked with the reduction of the risk of NJ. On the contrary, decreased probiotic intake was associated with augmented NJ risk.

### Exclusive colostrum feeding contributes to the reduction of NJ

We next interrogated the possible impact of different types of breastfeeding on NJ occurrence. There was a significant difference in the feeding patterns between the two groups (*P* < 0.001). Specifically, 73.0% of HC neonates received exclusive colostrum, compared to 45.4% in the NJ group. Exclusive formula feeding was comparable between the two groups (2.7 vs 3.4%). In contrast, mixed feeding was more prevalent among NJ infants (51.2% and 3.4%) than among HC infants (24.2% and 2.7%) (Table 2). Taking the significant difference in feeding type between groups in the descriptive comparison into account, we evaluated its association in the feeding pattern and found that exclusive colostrum feeding was associated with decreased risk of NJ (AOR 0.41 (0.28, 0.62), *P* < 0.001) compared to other feeding types (mixed or formula) (Table 3 part B). Taken together, our data suggest that exclusive colostrum feeding is an important behavioral practice strongly associated with reduced risk of NJ.

### Maternal probiotics intake and baby feeding type synergistically reduced NJ

Given the positive effects of both probiotic intake and breastfeeding method on NJ, we further investigated whether a potential synergistic effect and interaction existed between these two modifiable exposures. We found that among the exclusively colostrum-fed babies, increased probiotics intake pattern reduced the odds of NJ by 52% (AOR 0.48 (0.26 0.90), *P* = 0.022), and maintained intake by 85% (AOR 0.15 (0.06, 0.39), *P* < 0.001) in comparison with decreased intake (Fig. 2B). Similarly, among the mixed-fed group, increased and maintained probiotic intake during pregnancy, compared to decreased intake, showed a similar trend in lowering the risk of NJ, though the effect was not statistically significant (Fig. 2B). Our subgroup analysis was also adjusted for maternal employment, pre-labor nausea, PE, GDM, and birth weight, and no significant difference was demonstrated (Table 4). In addition, we further identified that multiparity was associated with reduced risk of NJ (AOR 0.43 (0.26, 0.72), *P* = 0.001). Conversely, intrapartum antibiotics use was associated with increased NJ risk by 94% (AOR 1.94 (1.19, 3.16), *P* = 0.008) (Table 4). These findings demonstrated that the increased maternal probiotics intake further lowered NJ occurrence in exclusive colostrum-fed and mixed-fed cases.

**Table 4:** Subgroup multivariable logistic regression analysis of probiotics intake pattern and baby feeding type

	Study variables		AOR (95% CI)	*P*-value
Exclusive colostrum fed	Parity	Primipara	Ref	Ref
		Multipara	0.43 (0.26, 0.72)	**0.001**
		Grand multipara	0.77 (0.28, 2.12)	0.609
	Prelabor nausea		1.45 (0.83, 2.54)	0.187
	Preeclampsia		1.92 (0.59, 6.24)	0.277
	Gestational diabetes		0.76 (0.17, 3.40)	0.715
	Intrapartum antibiotics use		1.94 (1.19, 3.16)	**0.008**
	Baby sex	Male	Ref	Ref
		Female	0.86 (0.53, 1.41)	0.555
Mixed fed	Maternal employment		0.49 (0.21, 1.18)	0.110
	Prelabor nausea		1.57 (0.65, 3.76)	0.316
	Preeclampsia		1.84 (0.61, 5.58)	0.281
	Gestational diabetes		1.04 (0.37, 2.96)	0.931
	Birth weight (gms)	< 2500	Ref	Ref
		2500–3999	0.36 (0.11, 1.12)	0.078
		≥ 4000	0.66 (0.15, 3.08)	0.606

*Ref* reference, exclusive colostrum feeding, feeding only BM in hospital (within 1–4 days after delivery); mixed feeding, feeding both BM and formula in hospital (within 1–4 days after delivery); *BM*, breast milk; *gms*, grams

## Discussion

By investigating the CHILD cohort with over 3000 paired mother-infant participants, we systematically evaluated the potential risk factors and preventive strategies for NJ. Although lack of detailed information regarding the specific type, dose, duration, and indication of maternal probiotic intake, our data suggested that mothers who increased and maintained their probiotic intake throughout pregnancy, compared to their pre-pregnancy levels, exhibited 47% and 56% lower odds of NJ sequentially than those who decreased their intake, despite the type of baby feeding. Compared to mixed-feeding, exclusive colostrum feeding also reduced the risk of NJ by 59%. Moreover, term delivery and multiparity were also associated with reduced risk of NJ. Conversely, intrapartum antibiotic use, maternal pre-labor nausea, and pre-eclampsia were identified with the increased risk of NJ. Our results suggest that prenatal probiotic intervention and breastfeeding approach are promising modifiable factors to lower the risk of NJ.

Although it is still in debate for the etiology of NJ, including hemolytic diseases, liver dysfunction, and infection [40, 41], accumulating evidence suggests that the infant gut microbiome plays a vital function in the NJ progression [42]. Bilirubin overaccumulation is the root physiology of NJ. The catabolism of conjugated bilirubin into stercobilin and urobilin is mainly regulated by the intestinal microbiome [43]. Impairment of this process leads to the deconjugation and enterohepatic reabsorption of bilirubin [30, 31], consequently causing hyperbilirubinemia [44]. Recently, a study uncovered that BilR is a major bacterial enzyme involved in bilirubin clearance, which is enriched in the *Firmicutes* bacteria [32]. Conversely, microbial-sourced β-glucuronidase deconjugates bilirubin and facilitates enterohepatic recirculation [30, 31], thereby leading to hyperbilirubinemia [44]. Therefore, it is a feasible approach for NJ prevention by targeting the microbiome.

Breastfeeding is a process of nourishing infants with breast milk, which is an essential natural food rich in nutrients and antibodies [45]. It has tremendous benefits beyond nutrition, such as immune development and protection, cognitive and digestive maturation and integrity, and reduced risk of disease [45, 46]. We found that exclusive colostrum feeding largely leads to a lower NJ risk (by 66%), compared to non-exclusive feeding. Our results are in line with the known effects of exclusively breastfeeding in fostering neonatal gut microbiome health [35]. Importantly, these findings provide additional supportive evidence that reinforces the World Health Organization’s (WHO) recommendations for early initiation of breastfeeding and continuing exclusive breastfeeding for the first 6 months [47].

Probiotic supplements have exerted substantial benefits in remodeling human gut-microbiome composition, enhancing immunity, inhibiting pathogens, improving digestion, and producing beneficial metabolites [48, 49]. During pregnancy, maternal probiotics intake reduces the risks of gestational diabetes, rectovaginal group B streptococcus colonization, and lactational mastitis through reshaping gut microbiome composition and diversity [21]. It also affects the infant’s gut microbiome and health [26]. Lines of evidence suggest that infants of mothers with probiotic intake during gestation have greater gut microbiome diversity and higher abundances of beneficial bacteria. Moreover, it improved infant gut health, including gastrointestinal symptoms and colic [26, 50]. In our study, we provide a novel benefit of maternal probiotic intake on its preventive association with NJ. Besides its effects on remodeling infant gut microbiome homeostasis, maternal probiotics intake also increases defecation frequency in infants, thereby promoting the excretion of bilirubin and reducing the enterohepatic re-circulation of unconjugated bilirubin [51], which may collectively improve neonatal bilirubin metabolism [32].

Importantly, our study further reveals a potential synergized interaction between maternal probiotics intake and exclusive colostrum feeding on mitigating the NJ by subgroup analysis. Significant NJ risk reductions were observed among participants who increased (47%) and maintained (56%) probiotics intake during pregnancy in exclusive colostrum-fed neonates. This implies that breast milk, particularly colostrum, has a great potential for mediation between mother and baby for microbiome crosstalk after maternal probiotics intake and reduces the risk of NJ. A similar trend was observed in the mixed-fed group. Growing evidence has suggested that maternal probiotic intake in late gestation can enrich beneficial bacteria in the BM microbiome, reduce inflammatory markers such as interleukin-6 and c-reactive protein, modulate human milk oligosaccharide composition, and thereby amplify the protective effects of breastfeeding [26, 52–56]. Of interest, some of the recent infant formulas have incorporated probiotic ingredients [57], which may represent an alternative avenue to directly shape the gut ecosystem in newborns. However, its potential effects on NJ warrant rigorous investigation.

Moreover, several potential risk factors for NJ were identified in our study. For instance, maternal intrapartum antibiotic use has been shown to increase the risk of NJ, which is associated with infant gut dysbiosis [58]. Meanwhile, pregnancy complications such as PE and pre-labor nausea were associated with the increased odds of NJ. Preeclampsia is also reported with maternal gut microbiome dysbiosis [59]. Interestingly, maternal probiotics intake can improve gut dysbiosis and prevent associated complications [60].

This study possesses notable strengths. First, the CHILD cohort is a large-scale, prospective, and population-based longitudinal birth cohort with a rigorous design. The well-documented maternal and neonatal clinical information enabled us to comprehensively investigate the potential protective or risk factors for NJ, while adequately adjusting for confounders. Moreover, our analysis incorporated a large sample of over 3000 mother-infant pairs, which substantially enhanced statistical power and improved the generalizability and robustness of our findings. However, several limitations are fully acknowledged in our study. First, with the CHLID cohort, the questionnaire-based data on maternal probiotic intake lacked details on dose, duration, and indication. Meanwhile, it did not clearly specify whether probiotic intake referred exclusively to food-based sources (e.g., yogurt) or included probiotic supplements. Likewise, the information on the mode of colostrum feeding (i.e., direct breastfeeding vs pumped milk) was not available. Moreover, in the subgroup analysis, the regression model could not be fitted for the “formula-fed” group due to an insufficient number of participants in this category. Of note, the identification of potential risk factors for NJ in our study was constrained by the data available within the CHILD cohort. In addition, our conclusions are based on the results of observational associations, implying the need for further research to examine the compositional alterations in the maternal gut microbiome, the BM microbiomes, and the infant gut microbiome to elucidate the underlying mechanism. Further randomized controlled trials are needed to assess the preventive impact of maternal probiotic use and breastfeeding types on neonatal jaundice risk.

## Conclusions

By leveraging the CHILD longitudinal birth cohort study data, we assessed the association between maternal probiotic use and exclusive colostrum feeding with NJ. We found that maternal probiotics intake during pregnancy and breastfeeding were associated with the risk of NJ. In particular, increasing and maintaining the intake of probiotics during gestation were associated with reduced odds of NJ. Moreover, exclusive colostrum-feeding also contributes to a lower NJ risk. Our findings also emerge as modifiable strategies for NJ prevention. Importantly, our data advocates for integrating exclusive colostrum feeding and maternal probiotic intake in perinatal nutrition counseling and neonatal care to minimize the risk of NJ. Further studies are warranted to evaluate whether maternal probiotics during pregnancy effectively reduce NJ beyond the association.

## Supplementary Information

Additional file 1: Table S1. Terminologies and definitions of probiotic intake pattern, feeding mode, and neonatal jaundice in the CHLID cohort. Table S2. Data cleaning and statistical methods for model-building procedures.

## Data Availability

The data were exported from the CHILD database (childstudy.ca), and the datasets used and/or analyzed during the current study are available from the corresponding author on reasonable request.

## Abbreviations

AOR: Adjusted odds ratio
BM: Breast milk;
CHILD: Canadian Healthy Infant Longitudinal Development cohort study
CI: Confidence interval
NJ: Neonatal jaundice
GDM: Gestational diabetes
PE: Preeclampsia
ECF: Exclusive colostrum feeding
